# Prevention of Morbidity in Sickle Cell Disease (POMS2a)—overnight auto-adjusting continuous positive airway pressure compared with nocturnal oxygen therapy: a randomised crossover pilot study examining patient preference and safety in adults and children

**DOI:** 10.1186/s13063-019-3461-x

**Published:** 2019-07-18

**Authors:** Jo Howard, Sophie A. Lee, Baba Inusa, Man Ying Edith Cheng, Cheema Bavenjit, Isabel C. Reading, Sally Ann Wakeford, Johanna C. Gavlak, Patrick B. Murphy, Nicholas Hart, Atul Gupta, Sati Sahota, Eufemia Jacob, Maria Chorozoglou, Carol Ossai, Maureen Gwam, Fenella J. Kirkham, Angela M. Wade, Christina Liossi

**Affiliations:** 1 Department of Haematology, Guy’s and St Thomas’ Hospitals NHS Foundation Trust, London, UK; 2 Centre for Applied Statistics Courses, UCL Great Ormond Street Institute of Child Health, London, UK; 3 Evelina Children’s Hospital, Guy’s and St Thomas’ Hospitals NHS Foundation Trust, London, UK; 4 Research Design Service, University Hospital Southampton, Southampton, UK; 5 British Columbia Children’s Hospital, Vancouver, Canada; 6 Department of Child Health, University Hospital Southampton, Southampton, UK; 7 Developmental Neurosciences Section and NIHR Biomedical Research Centre, UCL Great Ormond Street Institute of Child Health, 30 Guilford Street, London, WC1N 1EH UK; 8 King’s College London, London, UK; 9 Lane Fox Respiratory Unit, Guy’s and St Thomas’ Hospitals NHS Foundation Trust, London, UK; 10 School of Nursing, University of California Los Angeles, Los Angeles, CA USA; 11 Clinical and Experimental Sciences, Faculty of Medicine, University of Southampton, Southampton, UK; 12 Sickle Cell and Young Stroke Survivors Charity, London, UK; 13 Department of Psychology, University of Southampton, Southampton, UK; 14 Department of Psychology, Great Ormond Street Hospital for Children NHS Foundation Trust, London, UK

**Keywords:** Sickle cell anaemia, Inherited diseases, Haemoglobin, Qualitative method, Statistical method, Randomised crossover trial

## Abstract

**Design:**

This randomised crossover trial compared nocturnal auto-adjusting continuous positive airway pressure (APAP) and nocturnal oxygen therapy (NOT) in adults and children with sickle cell anaemia, with patient acceptability as the primary outcome. Secondary outcomes included pulmonary physiology (adults), safety, and daily pain during interventions and washout documented using tablet technology.

**Methods:**

Inclusion criteria were age > 8 years and the ability to use an iPad to collect daily pain data. Trial participation was 4 weeks; week 1 involved baseline data collection and week 3 was a washout between interventions, which were administered for 7 days each during weeks 2 and 4 in a randomised order. Qualitative interviews were transcribed verbatim and analysed for content using a funnelling technique, starting generally and then gaining more detailed information on the experience of both interventions. Safety data included routine haematology and median pain days between each period. Missing pain day values were replaced using multiple imputation.

**Results:**

Ten adults (three female, median age 30.2 years, range 18–51.5 years) and eleven children (five female, median age 12 years, range 8.7–16.9 years) enrolled. Nine adults and seven children completed interviews. Qualitative data revealed that the APAP machine was smaller, easier to handle, and less noisy. Of 16 participants, 10 preferred APAP (62.5%, 95% confidence interval (CI) 38.6–81.5%). Haemoglobin decreased from baseline on APAP and NOT (mean difference −3.2 g/L (95% CI −6.0 to −0.2 g/L) and −2.5 g/L (95% CI −4.6 to 0.3 g/L), respectively), but there was no significant difference between interventions (NOT versus APAP, 1.1 (−1.2 to 3.6)). Pulmonary function changed little. Compared with baseline, there were significant decreases in the median number of pain days (1.58 for APAP and 1.71 for NOT) but no significant difference comparing washout with baseline. After adjustment for carry-over and period effects, there was a non-significant median difference of 0.143 (95% CI −0.116 to 0.401) days additional pain with APAP compared with NOT.

**Conclusion:**

In view of the point estimate of patient preference for APAP, and no difference in haematology or pulmonary function or evidence that pain was worse during or in washout after APAP, it was decided to proceed with a Phase II trial of 6 months APAP versus standard care with further safety monitoring for bone marrow suppression and pain.

**Trial registration:**

ISRCTN46078697. Registered on 18 July 2014

## Introduction

There is a high prevalence of obstructive sleep apnoea (OSA) [1] and overnight oxygen desaturation [2] in children and adults [3] with sickle cell anaemia (SCA, HbSS, or HbSβ 0-thalassaemia). In the general population, continuous positive airway pressure (CPAP) is an established treatment for OSA in adults and children [4–6], and oxygen supplementation plays a role in the management of chronic obstructive pulmonary disease [7, 8] in adults and of bronchopulmonary dysplasia in children, respectively. However, there are few data on the acceptability or efficacy of either method of respiratory support in SCA. This is in part due to concerns about the safety of oxygen administration in this condition, specifically with respect to bone marrow suppression and rebound pain [9–11]. Optimal management of nocturnal desaturation therefore remains controversial, and the possibility that reducing exposure to hypoxia might reduce SCA complications has received little attention.

Auto-adjusting continuous positive airway pressure (APAP), which increases airway pressure when the patient’s airway obstructs, appears to improve attention/processing speed, measured as cancellation, and reduce pain episodes in children with SCA [12]. CPAP has been used peri-operatively in adults with sickle cell disease (SCD) [13] but, although the results of a Phase II study are awaited [14], there are as yet no published data on longer term use in adults. However, there is observational evidence that nocturnal oxygen therapy (NOT), used for a period of at least 6 months in adults with SCA and severe nocturnal hypoxia, is safe and easy to use [11]. It is not known whether patients would prefer one of these treatments over the other, an essential prerequisite for a two-arm Phase II trial comparing either method of overnight respiratory support with standard treatment [14].

The Prevention of Morbidity in Sickle Cell Disease (POMS2a) study is a National Institute for Health Research (NIHR) Research for Patient Benefit (RfPB) funded pilot crossover trial to compare APAP with NOT in adults and children with SCA to identify: 1) the treatment strategy most acceptable to patients and families for further investigation; 2) whether there are likely short-term safety concerns (e.g. worsening anaemia or pain), or physiological benefits (e.g. pulmonary function) or clinical benefits (e.g. reduced pain) for overnight oxygen therapy or APAP; 3) the feasibility of completing a pain diary as an app on an iPad to collect daily information on site and severity of pain; and 4) the main cost drivers and potential cost implications of providing the intervention [15].

## Methods

### Inclusion and exclusion criteria

**Fig. 1:**
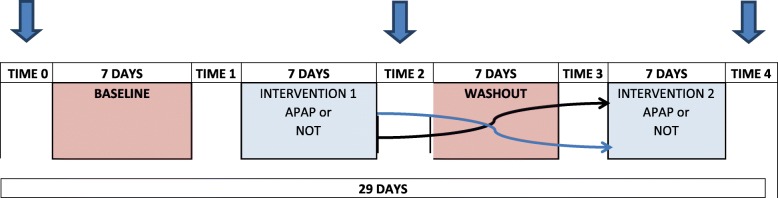
Design of the POMS2a randomised crossover study of auto-adjusting continuous positive airways pressure (APAP) and nocturnal oxygen therapy (NOT) in sickle cell anaemia

This was a randomised crossover trial of APAP and NOT (Fig. 1) conducted at Guy’s and St Thomas’ Hospital NHS Foundation Trust [15]. Inclusion criteria included: age over 8 years, a diagnosis of SCA, the ability to speak English, gave informed consent or assent, and could use an iPad. Exclusion criteria included: current or prior experience with overnight respiratory support, hospital admission for acute sickle complications within the past 1 month, > 6 admissions for acute sickle complications within the past 12 months, existing respiratory failure, decompensated cardiac failure, pregnancy, and contraindications to APAP [15].

### Baseline data

Baseline haematology, oximetry, medical, and quality of life (PedsQL) [16, 17] questionnaires and 1 week of baseline pain data were obtained. Overnight oximetry was measured at home over two nights at baseline to document mean and minimum overnight oxygen saturation, but evidence of overnight hypoxia and/or OSA was not necessary for inclusion in the trial.

### Randomisation and blinding

Patients were randomly assigned to receive APAP or NOT for 1 week, followed by 1 week washout and then 1 week of the alternative intervention. In view of previous concerns about safety [9, 10], which the children might not have been able to articulate, the adult participants were randomised first. Variable block randomisation of the order of treatment (APAP/NOT) was undertaken by an independent statistician at the University of Southampton. It was not possible to blind the participant, local Principal Investigators, study co-ordinator, sleep physiologist, or psychologist to the order of treatment. However, the Chief Investigator, statistician, and technician performing spirometry, i.e. those responsible for documenting the quantitative endpoints, were blinded to which intervention was given in which order.

### Intervention

The REMstar® Auto System (Philips Respironics) is an APAP device designed for the treatment of OSA. When set in the APAP mode, the system monitors breathing whilst sleeping and automatically adjusts the pressure to overcome upper airway obstruction. APAP was set at 4 cmH_2_O with an upper limit of 10 cmH_2_O administered via a nasal or oral-nasal mask. For NOT, oxygen concentrators (Philips Respironics) were used to administer oxygen therapy at 0.5 L/min in children and 1 L/min in adults via nasal cannula or mask depending on participant preference.

### Compliance

Support from a respiratory physiologist with experience of APAP and NOT was available to initiate and maximize compliance with the interventions. For the APAP intervention, compliance with treatment was formally assessed using Encore Pro™ data management software via a SmartCard that recorded both qualitative and quantitative data on a single card collected after the 7-day intervention period. For the oxygen therapy, the sleep physiologist telephoned participants 24 h after starting each intervention and mid-treatment and kept a record of telephone support during the study.

### Primary outcome: patient preference

The primary outcome was patient preference assessed via semi-structured interviews that explored participant experience with each intervention and the electronic pain diary app.

### Semi-structured interview guide

All qualitative interviews were conducted using a semi-structured interview guide. The interview guide was structured using a funnelling technique, which started with a general question about prior experience and expectations of different treatments and then worked towards gaining detailed information about the experience of using the interventions. Initial probing questions were open ended, such as “Can you tell me more about that?” Additional probing questions asked about different types of influences including people at home, environmental factors such as space, and others. On principle, neither the questions nor analysis were directed by theory. At the end of the interview, the participant was asked if there was any other information that would be important in understanding her or his treatment choice. The interview guide evolved throughout the course of the study to explore developing concepts; however, each participant was asked about the same main focal areas to ensure dependability in the data.

### Data collection

After participants were re-consented over the telephone, the interview guide was followed with flexibility for the informant’s pace, comfort, and expression. Field notes about the interaction were transcribed immediately following the telephone call.

After transcribing the interviews with participants who had completed both interventions, content analysis was used to analyse the data. Content analysis is the process of subjective interpretation of text data through systematic classification of coding and identifying themes and patterns [18]. Specifically, this dataset was examined using conventional content analysis, a method that is used to describe a phenomenon by allowing codes to flow from data and is focused on the surface meaning on the words being analysed. Through this methodology, codes are developed after reading and re-reading the text for 3–4 transcripts and then applied to the rest of the data. If the new data does not fit into a previous code, a new code is developed and, finally, codes are assimilated based on similarity [18].

Content analysis was deemed appropriate due to the quantitative and qualitative nature of the data. Both open-ended and close-ended questions were used to ascertain patient feasibility, acceptability, and preference. Through content analysis, both the frequency of a specific response (i.e. preference for APAP over NOT) could be counted and participant anecdotes could be noted. This gives an objective method to quantify responses, as well as derive anecdotal meaning behind the numbers. A conventional method was used as opposed to a directed or summative method due to the limited background on the usage of APAP (or any form of overnight respiratory support) in SCA patients [19].

The content analysis began by the researcher reading through the transcript as a whole and then returning to focus on the first four transcribed interviews. These four transcribed interviews were read thoroughly and keywords describing answers to the semi-structured interview questions were highlighted. After highlighting the transcripts, the keywords were sorted into codes that encompassed similar themes. Thereafter, the codes were further sorted under categories and subcategories to create an organized and systematic way to consolidate and analyse the data. For example, three main categories were created to separate attitudes prior to treatment, experiences during treatment, and post-treatment thoughts. After creating a master list of codes for both adults and children, the remaining transcripts were highlighted and then transferred into the coding sheets. Wherever a question evoked a response that did not fit into the coding sheet, it was noted and added as miscellaneous until a relevant code was created. In the final step, the master list of codes was re-evaluated to see that codes were exhaustive, mutually exclusive, and independent.

### Secondary endpoints

Secondary endpoints were focused on addressing concerns about safety, specifically the possibility of rebound pain during the washout period [9] and of bone marrow suppression during 1 week of treatment manifest as reduced erythropoietin, reticulocyte count, red cell count, haemoglobin and haematocrit levels [9], or of haemolysis manifest as an increase in bilirubin or lactate dehydrogenase [20].

In addition to documenting admissions for pain as severe adverse events, iPad mini-tablet technology [21] was used to collect daily pain scores for the week immediately before the first intervention, during the first intervention, during washout from the first intervention, and during the second intervention. The majority of the children managed the app by themselves but some of the younger children were assisted by their parents.

### Confidentiality

The paper data were stored in a locked cupboard and the electronic data were anonymised and stored on a password-protected computer.

### Sample size

This was a pilot trial [15] to determine patient preference for a Phase II study [14]. We wanted to include adults as well as children in this pilot since obstructive sleep apnoea and nocturnal oxygen desaturation have been described in all age groups but the only previous pilot trial included only 24 children [12]. It was not considered appropriate to include a power calculation, but we found no evidence for bone marrow suppression or rebound pain in 12 children on APAP in the previous pilot trial [12]. We therefore aimed to recruit around 10 adults and 10 children in this pilot crossover trial of two possible treatments to test the safety of the alternative interventions and the feasibility of running such a study [15] before beginning the Phase II trial [14]. Twenty patients (10 children and 10 adults) were considered sufficient for the qualitative interviews for the primary endpoint.

### Statistical analysis

Qualitative and quantitative data are reported according to the planned statistical analysis [15].

### Additional statistical analysis to account for missing data

The number of pain days reported was compared between periods (baseline, wash-out, and both treatment periods) using a bootstrap 95% confidence interval (CI) of the within-person median difference. This difference was given before and after adjusting for potential period and carry-over effects.

To overcome the problem of missing data, multiple imputation was used to impute the binary variable representing pain or no pain; 100 imputations were carried out. The package ‘mi’ was used in R [22], and the variables included in the model were subject ID, day of treatment, and intervention. As the variable of interest (number of pain days within a week) was not normally distributed, bootstrapping [23] was used with 10,000 iterations to estimate the distribution of median pain days, which was normally distributed around the median. The mean and standard deviation of the 100 imputed distributions were used to give estimates of the median and variance (calculated from the bootstrap standard error estimate and the sample size) of the within-imputation medians, which could then be combined using Rubin’s rules [24]. Note that this process, whilst giving the optimal estimates of the median numbers of pain days, does not necessarily yield integer values. A similar process was used to obtain 95% CIs for the differences between baseline and washout measures, treatment, period, and carry-over effects [25]. The intention of these analyses was not to determine whether one treatment was significantly better than the other but to gain information on the effect size necessary to inform a future adequately powered trial of treatment.

Sensitivity analyses considered the ‘best case scenario’ where all missing values were recorded as no pain, and the ‘worst case scenario’ with all missing values recorded as pain days.

## Results

### Participants

Thirteen adults were screened; three declined, citing upcoming holidays (*n* = 2) and inconvenience due to living with many relatives. Thirteen children were screened; one declined because of claustrophobia and unwillingness to use a mask and one was not recruited as the study had finished. Ten adults (three female, median age 30.2 years, range 18–51.5 years) and eleven children (five female, median age 12 years, range 8.7–16.9 years) were enrolled (Table 1). The additional child was recruited because a child did not tolerate the first intervention and withdrew within 24 h of a month-long study, precluding appropriate assessment of the primary or most secondary endpoints (see below).

**Table 1:** Patient characteristics

	Adults (*n* = 10)	Children (*n* = 11)
Male, *n* (%)	7 (70%)	6 (55%)
Receiving hydroxyurea, *n* (%)	5 (50%)	6 (55%)
Interviewed, *n* (%)	9 (90%)	7 (64%)
Nadir SpO_2_ < 2 standards below mean for age for people with RDI < 5 [26], *n* (%)	9 (90%)	9 (82%)
Mean SpO_2_ < 10th centile (96% [27]), *n* (%)	9 (90%)	9 (82%)
Nadir SpO_2_ < 10th centile (93.5% [27]), *n* (%)	10 (100%)	11 (100%)
Nadir SpO_2_ < 2.5th centile (85.2% [28]), *n* (%)	8 (80%)	9 (82%)
Nadir SpO_2_ < 2 standards below mean (86.8% [29]) , *n* (%)	9 (90%)	9 (82%)
Age (years), median (range)	30.2 (18–51.5)	12 (8.7–16.9)
Overnight oximetry over 2 nights, median (range)		
Mean SpO_2_ _Median (range)_	92.6 (87.7–97.3)	95.4 (85.0–97.7)
Nadir SpO_2 Median (range)_	79 (65.5–87.4)	81.1 (73.7–90.7)
APAP adherence, median (range)		
Nights used	6 (3–7)	7 (1–7)
Hours per night over 7 days	4.12 (0.55–6.77)	6.18 (0.75–9.19)
Hours per night over nights used	5.67 (1.27–7.83)	6.18 (2.79–9.19)

*APAP* auto-adjusting positive airway pressure, *RDI* Respiratory Disturbance Index, *SpO*_*2*_ oxygen saturation

### Oximetry at baseline

Compared with the normative data in children and adults undergoing overnight polysomnography and oximetry but with a Respiratory Disturbance Index (RDS) < 5 [26] and children in the asymptomatic general population undergoing overnight oximetry with or without polysomnography [27–29], mean and minimum overnight oxygen saturation (SpO_2_) before randomisation was abnormal in the majority of adults and children (Table 1). In the five adults on hydroxyurea to maximum tolerated dose, mean overnight SpO_2_ was lower (mean 92 ± 2.8% vs 93.2 ± 3.0%) but nadir SpO_2_ was slightly higher (78.9 ± 7.5% vs 76.4 ± 76.3%). The pattern was similar for children, with lower mean SpO_2_ (92.2 ± 4.5% vs 95.1 ± 2.6%) but slightly higher nadir SpO_2_ (78.6 ± 4.5% vs 77.7 ± 2.9%) in the six children on hydroxyurea. None of these differences were statistically significant.

### Quality of Life

Table 2 shows the results for the PedsQL quality of life measure at baseline. We used the validated sickle module for the children and the adult version of the PedsQL for the adults. For the children, the data from a large study of PedsQL in children with SCD [30] are shown for comparison. For most domains, scores appeared higher, i.e. better health-related quality of life, in the small sample of nine children completing the sickle module of the PedsQL in our study. Only two children and four adults completed follow-up PedsQL questionnaires (data not shown), citing lack of time at follow-up appointments.

**Table 2:** Quality of life at baseline

PedsQL sickle cell disease module scales	Number of items	This study					Panepinto et al. [30]				
		*n*	Mean	SD	% Floor	% Ceiling	*n*	Mean	SD	% Floor	% Ceiling
Child self-report											
SCD total score	43	9/11					240	62.4	18.6	0	0.6
Pain and hurt	9	9/11	81.2	21.4	0	11	243	66.7	20.9	0	5.3
Pain impact	10	9/11	71.4	24.4	0	11	239	54.0	24.8	0.3	5.6
Pain management and control	2	9/11	80.6	19.9	0	44	235	54.9	29.9	7.5	10.6
Worry I	5	9/11	71.1	19.6	0	11	240	63.5	26.2	0.9	10.0
Worry II	2	9/11	94.4	11.0	0	78	182	73.4	29.7	3.7	22.1
Emotions	2	8/11	54.7	35.9	13	25	238	62.0	33.1	8.4	19.3
Treatment	7	8/11	79.9	21.2	0	25	237	64.3	21.9	0.3	4.0
Communication I	3	8/11	76.0	21.6	0	37.5	239	73.8	24.9	0.3	23.4
Communication II	3	8/11	59.4	45.1	13	37.5	236	57.2	30.5	4.7	13.4
Adult core PedsQL											
Physical	8	10/10	64.4	26.8	0	0					
Psychosocial	15	10/10	75.2	18.3	0	0					
Emotion	5	10/10	74.0	19.3	0	20					
Social	5	10/10	77.5	21.5	0	20					
Work	5	10/10	74.0	19.4	0	10					

*SCD* sickle cell disease, *SD* standard deviation

### Qualitative endpoint

Detailed semi-structured qualitative interviews were completed for nine adults and seven children. The majority of participants (10/16; 62.5% (95% CI 38.6–81.5%)) showed a preference for APAP because the machine was smaller, easier to handle, and less noisy, and they preferred the idea that it would only correct breathing when abnormal. However, participants found the mask for APAP uncomfortable, although they noted that they slept better when using the APAP machine and were less fatigued (Table 3). Patients reported that NOT had a calming effect on them, but they also reported that it was very noisy and caused a dry nose, throat, and mouth (Table 3).

**Table 3:** Qualitative analysis

Overarching category	Question and response	Quotes
Semi-structured interview responses for children (*n* = 7)		
Attitudes prior to study	Reason(s) for participating • Doctor recommendation (1) • Personal reasons (1) • Research reasons (3) • Do not know (2)	“My son is out of breath every morning and his eye are always yellow”
	Prior knowledge about treatment • None (6) • Knowledge from previous experience (1)	“Sometimes when he has an asthma attack he uses the oxygen”
Experience of treatment	Cons of APAP or oxygen • Issues with machine apparatus (4) • Physiological side effects (1) • Uncomfortable sensations (1)	[APAP] “Sometimes it [the mask] keeps coming out and you have to put it back so it was just a bit problematic” [APAP] “Need to take it [the machine] off to breathe”
	Changes in symptoms or day-to-day life • None (4) • Physical differences (1) • Alertness/consciousness differences (3) • Sleep patterns: – (negative) (1)	[NOT] “…eyes was clear or very white… it wasn’t last for longer all day, but for a few hours”
Experiences with the machine	Difficulties using the machine • Yes (0) • No (7)	
Post-treatment thoughts	Most preferred machine • APAP (4) • NOT (3)	
	Advice for future users • Recommendation based on own preference (2)	
	Advice for researchers • Changes to machines (1) • Changes to protocol	[APAP] “Some people don’t have much space, so to make it secure would be hard”
Semi-structure interview responses for adults (*n* = 9)		
Attitudes prior to study	Reason(s) for participating • Doctor recommendation (3) • Personal reasons (2) • Research reasons (1) • Help others (1) • Personal and research (2) • Personal and help others (2) • Do not know (1)	“Benefit me now or in the future and other sickle patients… thought the trial could possibly help in the future if I need it or somebody else needed that kind of treatment.” “Always willing to help with the advancement of medical research” “I would really and truly hope it would benefit the sicklers behind me…Cause for myself.. I think I am long over the age now” “for the first time I was going to get/receive something in return for doing the study”
	Prior knowledge about treatment • None (7) • Knowledge from previous experience (2)	“I know oxygen calms you down when you take injections and stuff like that”
	Expectations of study • None (5) • Help with sickle cell symptoms (3) • Help with sleep (2)	“Oxygen levels would be better and help manage my pain” “Figure they [the machines] would help me during the night”
	Concerns of study • None (10) • Health (0) • Social (1)	“My concern is that it [the APAP machine] would fall and wake everyone”
Experience of treatment	Overall experience • Indifferent (2) • Positive (5) • Negative (2)	
	Pros of APAP or oxygen • Physiological factors (2) • Technical factors (7): - APAP (5) - Oxygen (2) • Overall mood (4)	[APAP] “It was a bit more intelligent in regards to... it would compensate for any air loss that you was breathing in” [APAP and NOT] “made me feel more relaxed” [NOT] “…especially at winter, in the night when you feel funny and your breathing is funny. It’s when you have it in the bedroom before the ambulance comes. That equipment, you can put in your mouth.. and that will help you oxygen levels as well before the ambulance arrives…and then they can give you more assistance to get to the hospital”.
	Cons of APAP or oxygen • Issues with machine apparatus (11) - APAP (6) - Oxygen (7) • Physiological side effects (3) - APAP (1) - Oxygen (2) • Uncomfortable sensations (1) • Social factors (1)	[APAP] “The mask over my face is a bit restrictive for moving around” [NOT] “The noise the machine made was louder” [APAP] “Made my throat a bit dry and it felt like… it feels that way… like I am catching a cold” [APAP] “I felt like I was suffocating” “I think it was just in terms of a family getting used to the machine”
	Changes in symptoms or day-to-day life • None (4) • Physical differences (5) - Positive: (4) - Negative: (1) • Alertness/ consciousness differences (6) - Positive: (5) - Negative (1) • Sleep patterns (13) - Positive: (10) - Negative: (3) • Eating pattern (1)	[APAP and NOT] “I didn’t have as many headaches [in the morning]” [APAP and NOT] “I wasn’t tired during the day” [NOT] “It felt when the room was hot and stuffy, I was still able to get a good oxygen supply”. [APAP and NOT] “I was able to get more sleep and uninterrupted sleep. Sometimes without the machine I will wake up in the middle of the night because my mouth is dry or something like that. With the machine, none of that happened”. [APAP] “Sometimes I felt like I couldn’t sleep through the night completely… But as the days got by, I got used to it.”
Experience with the machine	Difficulties in using the machine • Yes (0) • No (9)	[APAP] “Very easy. Easy to manage. Light machine… no problems” [NOT] “No it’s a very simple machine. You just press the button once, press it again and you configure it once and it stays like that”
Post-treatment thoughts	Most preferred machine • APAP (6) • Oxygen (3)	“The mask is more comfortable to sleep [APAP] with at night. With the other one [NOT] the mask is too big... it was hard for me to sleep.”
	Advice for future users • Recommendation based on own preference (7) • None (2)	
	Advice for researchers • Related to machines (5) • Related to protocol (2) • None (2)	“Interface for pain diary is primitive” “[make the mask] more comfortable to wear and sleep with at night.” [NOT] “Would take a lot longer to get used to”
	More questions • About questionnaire (1) • Related to machines (1)	
Overall experience with pain diary	• Positive (9) • Other (2)	“If I was having a bad day, it did ask more questions but I think that was fine… the way it is” “the interface… the actual thing that you fill out.. it does look slightly… primitive I would say….it doesn’t really matter realistically… but you want a nicer interface” “I had no incentive to fill in the pain diary when I was pain-free”

*APAP* auto-adjusting continuous positive airway pressure, *NOT* nocturnal oxygen therapy

### Serious adverse events

Two patients (one child; 9.5% (95% CI 3–29%)) were admitted with pain whilst in the APAP arm and were withdrawn from the trial. The adult completed 3 days of APAP at an average of 7.61 h and was admitted 2 days later with a painful crisis and subsequent acute chest syndrome. The child who did not tolerate APAP, and who stopped after using it for 5.26 h on 1 night, was admitted with a painful crisis 3 days later. No patients were admitted with pain whilst in the NOT arm but one adult (5% (95% CI 1–23%)) developed a pain crisis on day 2 of NOT which was self-managed at home.

### Haematological and biochemical safety

Haemoglobin and red blood cell count decreased after both interventions with similar mean differences (Table 4). One patient showed a decrease in absolute reticulocyte count from 128 × 10^9^/L to 53 × 10^9^/L at the end of NOT therapy, with no change in haemoglobin. Haemoglobin less than 60 g/L was not seen and there were no significant differences in reticulocyte count or erythropoietin, or in creatinine, the albumin:creatinine ratio, bilirubin, or lactate dehydrogenase levels after either intervention compared with baseline (Table 4).

**Table 4:** Clinical, haematological, biochemical, and pulmonary function values at baseline and after 1 week of overnight auto-adjusting continuous positive airway pressure (APAP) and nocturnal oxygen therapy (NOT) in patients with sickle cell disease

	*N* at baseline	*N* after 1st intervention	*N* after 2nd intervention	Baseline		APAP compared with baseline			NOT compared with baseline			NOT compared with APAP		
				Mean	SD	Mean difference	Lower 95% CI	Upper 95% CI	Mean difference	Lower 95% CI	Upper 95% CI	Mean difference	Lower 95% CI	Upper 95% CI
Heart rate	21	20	19	76	27	3.2	0.1	7.0	−0.6	−11.0	7.0	0.3	−2.3	3.4
Respiratory rate	21	20	19	17.9	2.0	−0.2	−0.6	0.2	−0.2	−1.4	0.5	−0.1	−1.0	0.6
Systolic blood pressure	21	20	19	120	13	−1.9	−5.2	1.3	−1.7	−5.1	1.6	0.5	−2.0	3.2
Diastolic blood pressure	21	20	19	63.6	10.6	2.0	−0.2	4.4	1.6	−0.9	3.9	−0.3	−2.6	1.9
Mean arterial blood pressure	21	20	19	100.9	11.2	0.7	−1.2	2.7	0.5	−1.5	2.3	0	−1.8	1.6
Pulse pressure	21	20	19	56.0	12.2	4.0	−0.4	8.3	−3.2	−6.9	0.9	0.8	−2.7	4.2
Daytime oxygen saturation	21	20	19	97.7	1.2	0.2	−0.3	0.6	0.3	−0.1	0.6	2.6	0.4	5.0
White blood cell count	21	21	20	9.6	3.0	1.0	−0.4	2.6	0.1	−0.8	0.9	−1.0	−2.8	0.5
Haemoglobin	21	21	20	86.6	13.1	−3.2	−6.0	−0.2	−2.5	−4.6	0.3	1.1	−1.2	3.6
Red cell count	21	21	20	2.9	0.7	−0.2	−0.3	0	−0.1	−0.2	0	0.1	−0.02	0.2
Absolute reticulocytes	20	20	19	194	77	−23.8	−78.8	21.3	−31.1	−70.3	0.5	−16.7	−51.2	19.3
Lactate dehydrogenase	19	16	17	505	150	20.6	−15.6	56.6	3.8	−35.9	42.5	12.1	−16.2	42.1
Alanine transferase	19	15	17	32	38	5.6	0.3	12.8	6.4	0.9	17.4	−1.6	−7.5	4.0
Erythropoietin	16	13	10	99	68	−20.0	−63.3	31.1	−27.5	−62.0	2.6	−4.2	−29.7	21.3
Creatinine	20	19	18	57.4	16.7	0.5	−2.9	4.3	2.1	−0.9	5.3	1.3	−0.2	4.7
Bilirubin	21	19	19	50	42	3.0	−2.8	9.1	−1.3	−7.1	3.8	−2.0	−6.9	2.6
Albumin creatinine ratio	10	9	9	2.6	2.2	−1.5	−5.3	0.5	−1.3	−4.2	0.3	−0.2	−1.4	1.2
Vital capacity (L)	10	9	9	3.7	0.9	0.1	0	0.2	0	−0.1	0.1	−0.1	−0.2	0.1
Vital capacity (%)	10	9	9	90.5	11.4	1.1	−4.3	5.4	−0.8	−3.0	1.8	−1.9	−4.3	1.7
Functional residual capacity (L)	10	9	9	3.0	0.6	−0.1	−0.3	0.1	−0.1	−0.2	0	0	−0.1	0.1
Functional residual capacity (%)	10	9	9	94.7	9.5	−2.1	−7.1	3.6	−1.7	−5.5	1.6	0.4	−3.9	5.0
Inspiratory capacity (L)	10	9	9	2.5	0.6	−0.1	−0.4	0	−0.1	0.1	0.13	0.1	−0.1	0.4
Total lung capacity (L)	10	9	9	5.3	1.0	−0.7	−2.0	0.1	−0.2	−0.3	0	0.5	−0.3	1.9
Total lung capacity (%)	10	9	9	89.3	9.5	−2.7	−8.0	2.6	−1.0	−4.7	3.7	1.7	−3.4	7.0
Residual volume (L)	10	9	9	1.7	0.5	−0.2	−0.4	0	−0.1	−0.3	0.2	0.1	−0.1	0.4
Residual volume (%)	10	9	9	105	33	−15.6	−30.2	−0.9	−3.3	−15.0	11.0	12.2	−2.1	25.4
Residual volume:total lung capacity (L)	10	9	9	32.1	8.5	−2.4	−6.2	2.1	−8.4	−24.7	2.1	−6.0	−24.7	4.9
Forced vital capacity (FVC) over 1 s	10	9	9	3.7	1.0	0.19	0	0.5	0.1	−0.1	0.4	−0.1	−0.2	0
Forced expiratory volume (FEV) over 1 s	10	9	9	2.9	0.7	0	0	0.1	0	0	0.1	0	−0.1	0
FEV over 1 s (%)	10	9	9	84.0	10.3	0.4	−3.2	3.1	−0.6	−3.1	2.0	−1.0	−2.9	0.6
FEV:FVC (%)	10	9	9	82.7	11.8	−1.4	−5.2	2.1	2.8	−0.1	7.3	4.2	−0.3	9.3
Forced expiratory flow (FEF) 25–75% (L/s)	10	9	9	3.6	1.3	−0.4	−1.0	0.2	−0.2	−1.5	1.4	0.2	−1.2	1.4
FEF 50% (L/s)	10	9	9	3.6	1.1	−0.2	−0.5	0.1	0	−0.3	0.2	0.1	−0.2	0.4
FEF 75% (L/s)	10	9	9	2.9	2.5	2.5	1.0	4.3	−0.3	−1.2	0.2	−2.8	−4.5	−1.2
Peak expiratory flow (L/min)	10	9	9	433	72	−102	−218	−2.6	−69.7	−169	23.8	32.3	−32.9	138
Daytime oxygen saturation during LFTs (%)	10	9	9	93.9	2.7	0.3	−2.9	3.6	0.1	−2.3	2.1	−0.1	−1.9	1.7

*CI* confidence interval, *LFT* lung function tests, *SD* standard deviation

### Lung function

Lung volume measures did not increase on APAP and there was little change in lung function after 1 week of either APAP or NOT in the adults (Table 4).

### Adherence

APAP adherence data are presented in Table 1. Two-thirds of the patients (eight children and six adults) adhered to APAP for more than 5 h for 6 or 7 nights. Adherence was a little better in the children in terms of the number of hours per night, perhaps because they were not sharing a bed and were supervised by their parents. However, one child refused to use the APAP machine at all after a very short trial on the first night. One adult ceased use of the APAP machine on becoming unwell with a cold. Further details of the qualitative experiences of APAP and NOT treatments in the adults and children are given in Table 3.

### Pain

iPad data were available for 70.4% of days. Data were missing for a variety of reasons, for example patients unable to use the iPad or forgetting to record data. There was no indication that the missing pain days were directly related to the pain itself and so it was assumed for the imputation analyses that all missing values were either missing completely at random or missing at random. All patients were analysed on an intention-to-treat basis.

The median number of pain days per week were 2 for baseline, and 0 for washout, APAP, and NOT (number of observations reported were 112, 97, 99, and 106 respectively out of a potential 21 × 7 = 147 measures per period) (Table 5). Only two participants recorded on every day; others missed between 1 and 23 days. After imputation of missing pain days, these medians stayed at 2 per week for baseline and 0 per week for APAP, but increased to 1 for washout and NOT assessment times. The within-person difference in the median number of pain days between baseline and the washout period was non-significant (median difference 1.02 days (bootstrapped 95% CI −0.146 to 2.18)), and thus there was no evidence that a week was not sufficient time to return to baseline. Conclusions were similar for both sensitivity analyses. Both APAP and NOT were associated with a reduced number of median pain days when compared with baseline (1.58 and 1.71, respectively). Sensitivity analyses (best and worst case scenarios) showed the same direction of difference, but this was no longer significant (Table 6).

**Table 5:** Number of pain days

Data	Baseline	APAP	Washout	NOT
Raw	2 (1, 3)	0 (0, 0)	0 (0, 1)	0 (0, 1)
Multiple imputation (100 imputations)	2	0	1	1
Best case (all 0)	2 (1, 3)	0 (0, 0)	0 (0, 1)	0 (0, 1)
Worst case (all 1)	3 (3, 4)	2 (1, 5)	3 (1, 4)	4 (1, 6)

Values are shown as median (95% bootstrap confidence interval from 10,000 bootstraps)

*APAP* auto-adjusting continuous positive airway pressure, *NOT* nocturnal oxygen therapy

**Table 6:** Difference in the number of pain days between interventions

Method	Baseline – washout	Washout –APAP	Washout – NOT	Baseline – APAP	Baseline – NOT	APAP – NOT
Multiple imputation	1.02 (−0.146, 2.18)	−0.249 (−1.12, 0.536)	−0.244 (−1.23, 0.738)	1.58 (0.509, 2.65)	1.71 (0.791, 2.62)	−0.00569 (−0.385, 0.373)
Best case	1.00 (0, 2.00)	0.00 (0.00, 1.00)	0 (−1.00, 0.00)	1.00 (0.00, 2.00)	1.00 (0.00, 2.00)	0.00 (−1.00, 0.00)
Worst case	0.00 (0.00, 2.00)	0.00 (−1.00, 1.00)	0.00 (−1.00, 2.00)	1.00 (−1.00, 2.00)	2.00 (−1.00, 2.00)	0.00 (−1.00, 1.00)

Values are shown as median (95% confidence interval)

*APAP* auto-adjusting continuous positive airway pressure, *NOT* nocturnal oxygen therapy

The results above did not adjust for any carry-over or period effects. A further analysis was carried out to calculate these effects as well as an adjusted treatment effect to compare NOT and APAP; these results are displayed in Table 7.

**Table 7:** Carry-over, treatment, and period effects after adjusting for each other

Type	Carry-over	Treatment	Period
Multiple imputation	0.981 (−0.523, 2.48)	0.143 (−0.116, 0.401)	− 0.257 (− 0.520, 0.00658)
Best case	0.518 (− 1.78, 2.81)	−0.0227 (− 0.609, 0.563)	0.523 (− 0.0635, 1.109)
Worst case	−0.400 (−4.49, 3.69)	0.318 (− 0.850, 1.49)	−1.32 (−2.49, − 0.150)

Values are shown with confidence intervals

Auto-adjusting continuous positive airway pressure (APAP) was set as the reference treatment

The estimated carry-over effect was higher for NOT compared with APAP but was not statistically significant (median difference 0.98 (95% CI −0.52 to 2.48)). A non-significant period effect showed that there were on average 0.26 fewer (95% CI −0.52 to 0.001) pain days for the treatment given in the second period; the direction of this effect differed between best-case analysis and the multiple imputation and worst-case results. Adjustment for period and carry-over effects attenuated the difference in treatment regimes, giving a median decrease in pain days of 0.14 for APAP compared with NOT (95% CI −0.12 to 0.40). Hence, the results were not compatible with any meaningful difference in the number of pain days between the two treatments.

### Cost of the interventions

The cost of a standard oxygen concentrator is about three times that of an APAP machine (£1025 vs £330 in 2013). The APAP machine is light enough to take on holiday while a semi-portable oxygen concentrator on a trolley costs £2500. Consumables (masks, tubing) are about 10 times more expensive for APAP (£150 vs £15) but, if fitted correctly, an APAP mask may last for over a year, while oxygen masks and tubing may be discarded more frequently. One family using coins for electricity noticed that they were using more electricity during the week on NOT.

## Discussion

### Primary endpoint

This study shows that patients with SCA are willing, in principle, to participate in research involving overnight respiratory support, at least at the exploratory stage. Neither APAP or NOT is an ideal therapy from the patient’s point of view, but the inconvenience and discomfort of the mask essential for APAP is preferred to the noise of an oxygen concentrator, for which a mask is optional. Around 1 in 10 patients did not tolerate overnight respiratory support for 1 week but from the pain diaries there was no evidence for rebound pain in the washout period after 1 week of intervention. There was little evidence for clinically significant bone marrow suppression with either therapy. However, this was a very small pilot study and more data are needed.

Despite clear indications that overnight oxygen supplementation using CPAP, APAP, or oxygen therapy can help improve the quality and life expectancy for patients with OSA in the general population, adherence rates remain between 45 and 70% [31–34]. Considering the low usage rates in OSA patients, it is logical to assume that similar factors that prevent OSA patients from using CPAP or APAP would apply to patients with SCA. Our data agree with these adherence rates for APAP. However, five patients (24%) were not considered adherent to APAP and two withdrew because they were admitted to hospital after receiving APAP for 1 and 3 days, respectively. We were unable to obtain similar data for NOT usage as the NOT machines did not have the capability to download adherence data. Participants told the co-ordinator that they had used the concentrator over the short period of the intervention, but we were not able to document how compliant they had been. The use of oxygen cylinders is an alternative which would enable the amount used to be calculated but there are concerns over fire risk, and the cylinders are bulky in the domestic setting.

Many patients decide whether to use CPAP early on in the treatment period, usually within the first week of treatment [35, 36]. After 1 week of treatment exposure, cognitive factors such as self-efficacy and perceptions of disease risk and treatment outcome have been identified as significant independent predictors of CPAP use both in the short (i.e. 1 month) and long term (i.e. 6 months) [32, 37, 38]. However, cognitive perceptions influence CPAP use only within the context of knowledge of CPAP treatment and treatment use [39].

Various attempts have been made to identify factors that predict short- and long-term adherence; however, the data remain largely equivocal [34]. In general, across age groups, factors that influence or predict CPAP use include: a) disease and patient characteristics (e.g. age, socio-economic status), b) treatment titration procedure, c) technological device factors and side effects (e.g. mask, heated humidification), and d) psychological and social factors (disease- and treatment-specific knowledge, presence of bed partner, maternal education) [33].

In terms of technological device factors and side effects, the comfort of the mask features prominently as a factor that influences adherence in PAP machines [33, 40]. A systematic review by Saywer et al. [33] concluded that, although the mask is often a point of contention, there has not been in-depth research about its effect on adherence; however, a full mask overall is associated with lower adherence than a nasal tube for CPAP. However, one participant in the current study found the nasal tube unbearable and preferred the mask. Prashad et al. [40] concluded that, although almost all users of CPAP found the mask uncomfortable, patients who regularly used CPAP found that the benefits of the treatment outweighed the mask’s discomfort. In this current study, five adults and three children expressed discomfort with the mask, with one participant finding the comfort level something she could not bear. In terms of oxygen therapy, three adults expressed an annoyance for the nasal cannula falling out at various points in the night. One child said that this frequent disconnection kept him up all night, and the mother reciprocated this by claiming she had to tape the nasal cannula to prevent it from falling out. This concern, however, is not reflected in the current literature for oxygen therapy.

The difference in pressures between the oxygen therapy and APAP also plays a role in comfort, specifically in APAP. On one hand, researchers thought the lower overall pressure throughout the night helped facilitate a preference for APAP; however, another study [31] discussed that the high pressures (that would be implemented when the patient stops breathing) caused an unpleasant sensation. Both these thoughts were reflected in certain ways in the interviews from the POMS2a trial. Three participants claimed that they preferred the pressure in the APAP machine because “it felt like [they] were taking in more oxygen” compared with the nocturnal oxygen. On the other hand, one adult and one child expressed that the high pressure of the APAP machine at certain points made them feel like they were suffocating, thus causing unpleasant sensations. Lastly, the noise and size of the machines also played a role in patient preferences, specifically for oxygen therapy. Three adults found the noise disturbing to the point where they had trouble sleeping, or that it disturbed other family members. Overall, in terms of machine mechanics, the biggest disadvantage for APAP usage is an uncomfortable mask, whereas oxygen therapy’s mask/nasal cannula is unstable and also has low pressures that do not feel as useful to patients.

Physiologically, patients found both positive and negative effects. Oxygen therapy caused a dryness/coldness in the throat, skin, or nose for three adults, which also reflects an obstacle to adherence in individuals with chronic obstructive pulmonary disease (COPD) [37]. In contrast, in one child the oxygen helped clear yellowness of the eyes. The APAP treatment made patients feel as if their airways were being cleared, thus making it easier to breathe. Many participants also discussed a change in sleep patterns as a result of the treatment: 10 were positive changes, and 4 were negative changes. Many responses were given in a general context, instead of being therapy specific; however, for the specified responses, three participants found it easier to sleep with the APAP machine compared with the oxygen supply. However, in almost every case it took the patients a few days to adjust to the new sleeping routines (wearing a mask, sleeping in a certain way, and so on). Randerath et al. [31] also found that PAP machines improved rapid eye movement (REM) sleep and decreased daytime sleepiness after 6 weeks of use in OSA patients.

In terms of psychological and social factors, both using PAP and oxygen therapy, many studies discuss the importance of the patient’s self-efficacy and belief in their ability to use the treatment on a daily basis and also their expectations regarding the effects of treatment [32, 33, 37, 40, 41]. For example, when COPD patients felt that oxygen therapy was improving their day-to-day lives, they were more likely to adhere to the 15-h oxygen requirement [42], similar to the children with OSA offered CPAP by Parshad et al. [40]. In the POMS2a trial, when asked if the patients felt any changes in day-to-day symptoms, eight claimed to feel no different, with one participant only feeling better for 1 day after treatment and then returning to baseline. One participant felt that using the treatment was detrimental in her day-to-day life, primarily due to loss of sleep; however, the remaining participants claimed an improvement in one aspect or another: five participants felt physical differences (clearing headaches, improve breathing, and so on), and four participants felt improvements in alertness during the day. This is important to examine further since previous literature shows that a positive change in day-to-day life increases compliance with the treatment [37, 40].

The impact that a therapy has on the social interactions of a patient also affects the feasibility of a treatment. Two adults, who had families, expressed an inconvenience caused by the machines in the sleeping patterns of the family. Additionally, one child expressed that he was concerned he might knock over the APAP machine and thus cause others in the house to wake up. Family factors, inconvenience to others, and self-image with family and friends all play a role in compliance with oxygen therapy or PAP machines [32, 33, 37].

Fortunately, every participant in the current study showed no difficulty in learning how to operate either the APAP machine or oxygen concentrator, which increases the likelihood of compliance with the treatment.

In contrast to the wider literature, none of our patients or parents expressed any concerns for APAP or NOT. Commonly, when discussing oxygen therapy, many participants express a fear of becoming addicted to oxygen or of having side-effects from too much oxygen [7, 43]. This concern was largely absent from the current study, which may also be because most participants did not have any prior knowledge of the two therapies before their introduction to this study.

### Overnight oximetry at baseline

The majority of patients who enrolled in this study had mean and nadir oxygen saturations lower than that reported in people without sleep-disordered breathing in the general population [26–29]. There are few data on overnight oximetry in asymptomatic adults and the reported data may be for the median overnight oxygen saturation [26, 28, 29], precluding comparison with our mean overnight oxygen saturation data. Daytime SpO_2_ is lower in patients with SCA with lower haemoglobin F (HbF) [44], and hydroxyurea, which is typically associated with an increase in HbF, may increase daytime SpO_2_ [45]. In two children, hydroxyurea therapy was associated with improvement in OSA [46] but there are few data on any effect on overnight mean or nadir oxygen saturation. Our data do not suggest that overnight oxygen desaturation is cured by hydroxyurea use, but more data are needed.

### Quality of Life

Measurements of quality of life were feasible at baseline using the PedsQL but there were difficulties in obtaining repeat measures because of the length of time required to complete the questionnaires. For the Phase II trial we will ensure that patients fill in the relatively simple EuroQol quality of life questionnaires [47].

### Feasibility of using tablet technology

One of the aims of the trial was to establish the feasibility of tablet technology to collect daily pain data. Tablet technology was acceptable to patients with satisfactory levels of completion, but a simpler system might reduce the proportion of missing values.

### Safety

Although three patients experienced significant pain during overnight respiratory support, there was no evidence of rebound pain in the washout period after the 7-day application of APAP. Analysis of the number of pain days showed that, although both treatments were associated with a median reduction in the number of pain days, the data were not compatible with any clinically meaningful difference between the effects of APAP and NOT. There was some evidence for erythroid suppression with both APAP and NOT, but with no difference between them. The slight reduction in haemoglobin was not considered clinically significant and nor were the small changes in lung function in the adults. APAP is considerably cheaper, an important health economic consideration. However, the numbers were very small and safety will be an important endpoint in the planned Phase II trial.

In conclusion, the POMS2a trial shows results on feasibility, preference, and acceptance of APAP and oxygen therapy that are in line with previous literature for the same treatment used in other populations, with a few exceptions. SCA patients expressed concern over the mask type, machine noise, and ability to sleep during both the treatments; however, 10/16 of interviewed participants, a point estimate of 62.5%, claimed they would prefer to use APAP as opposed to NOT for overnight respiratory support. In view of the point estimate of patient preference for APAP, and no evidence that pain was worse during APAP or in washout after APAP, with no evidence of clinically significant bone marrow suppression, and improved ease of collection of adherence data and lower cost it was decided to proceed with a Phase II trial of 6 months therapy with APAP versus standard care to assess its effects on pain, quality of life, and cognitive function [14].

## Data Availability

The datasets used and/or analysed during the current study are available from the corresponding author on reasonable request.
